# Chronotropic index mediates the relationship between lower-limb loss level and 6-minute walk test performance

**DOI:** 10.3389/fspor.2026.1830289

**Published:** 2026-05-20

**Authors:** Samantha J. Stauffer, Ryan T. Pohlig, David G. Edwards, Frank B. Sarlo, John R. Horne, Elisa S. Arch, Jaclyn M. Sions

**Affiliations:** 1 Department of Physical Therapy, Delaware Limb Loss Studies, University of Delaware, STAR Campus, Newark, DE, United States; 2 Biostatistics Core, University of Delaware, Newark, DE, United States; 3 Department of Epidemiology, University of Delaware, STAR Tower, Newark, DE, United States; 4 Department of Kinesiology & Applied Physiology, University of Delaware, Newark, DE, United States; 5 Christiana Spine Center, Newark, DE, United States; 6 Independence Prosthetics-Orthotics, Inc, Newark, DE, United States

**Keywords:** amputees, cardiovascular deconditioning, exercise test, exercise tolerance, heart rate, walk test

## Abstract

**Introduction:**

Lower-limb loss (LLL) significantly impacts exercise capacity. Low heart rate, i.e., chronotropic, response to exercise is known to contribute to reduced exercise capacity in other clinical populations; associations between population-specific variables (e.g., LLL level), chronotropic response to activity, and exercise capacity among adults with LLL remain unknown.

**Methods:**

In this cross-sectional study, 130 community-dwelling adults with unilateral below-knee (*n* = 95; 58.5 ± 14.9 years-old, 74.7% male) or above-knee (*n* = 35; 54.0 ± 13.7 years-old; 68.6% male) LLL provided demographic and past medical history, including LLL-specific data. Vital signs were assessed at rest and after completing a 6-Minute Walk Test (6MWT). Chronotropic index, defined as the percent of heart rate reserve used, was calculated using the equation [(posttest heart rate—resting heart rate)/[208 – (0.7*age) – resting heart rate]]. Between-group differences in participant characteristics and cardiovascular response to the 6MWT were evaluated. Predictors of chronotropic index were identified using linear regression modeling; mediation modeling was used to determine if chronotropic index mediated the relationship between LLL level and 6MWT distance. Participants with above-knee LLL demonstrated significantly greater chronotropic indices (31.9% vs. 21.1%, *p* = 0.021) and walked shorter distances during the 6MWT (328.9 ± 112.6 meters vs. 400.5 ± 126.4 meters, *p* = 0.004). Chronotropic index was positively associated with 6MWT distance (*β* = 1.674, p < 0.001); LLL level significantly predicted chronotropic index (*β* = −9.929, *p* = 0.011). Per mediation analysis, chronotropic index partially mediated the relationship between LLL level and 6MWT distance (indirect effect = −16.616, 95% confidence interval: −35.057, −2.326). The final model explained 43.5% of the variance in 6MWT distance.

**Discussion:**

Findings suggest chronotropic response may be a physiological contributor to reduced exercise capacity in adults with LLL. Thus, chronotropic index may be a critical target for improving exercise capacity in adults with unilateral LLL.

## 1 Introduction

Of the more than 300,000 people who will experience lower-limb loss (LLL) this year, up to 50% are not expected to survive five years (1). Beyond perioperative complications, cardiovascular disease contributes notably to excess mortality among adults with LLL (2). In fact, individuals with LLL are nearly twice as likely to die of cardiovascular disease when compared to adults in the general population (3). Cardiovascular disease mortality among adults with LLL is often attributed to inactivity or preexisting comorbid conditions that contributed to the need for amputation. However, heightened cardiovascular disease risk persists even when no differences in risk factors, such as physical activity, blood pressure, age, or smoking status, are observed (2, 4). Rather, physiological adaptation following LLL may drive elevated cardiovascular disease risk post-LLL.

Following LLL, systemic circulation is affected, potentially leading to maladaptive changes in the cardiovascular system over time. Removal of lower extremity musculature and a reduction in contractility of remaining musculature negatively affects skeletal muscle pump function (5), reducing venous return, cardiac preload, and stroke volume (6). To compensate, adults with LLL rely on increased heart rate (HR) to drive cardiac output, a response that is exaggerated with greater limb involvement, (i.e., higher level and/or bilateral amputations) (6). Increased HR and cardiac contractility are achieved via sympathetic nervous system activation and parasympathetic nervous system withdrawal (7). However, long-term sympathetic overactivity leads to adverse cardiovascular health outcomes, such as hypertension, left ventricular hypertrophy, and heart failure (8). Clinically, sympathetic overactivity may manifest as exercise intolerance (9, 10), as sympathetic overactivity disrupts the body's ability to adapt to activity-induced hemodynamic challenge. Thus, investigations into the association between cardiac response to activity and exercise capacity following LLL are necessary as a first step in identifying treatment pathways for LLL-associated exercise intolerance.

Chronotropic incompetence, defined as insufficient increase in HR to meet activity demands, is predictive of cardiovascular disease mortality, even after adjusting for factors such as age, diabetes, hypertension, hypercholesterolemia, fitness level, and smoking (11). Chronotropic incompetence is a primary determinant of exercise intolerance, as HR is the strongest contributor to aerobic exercise sustainability (11, 12). Low chronotropic index, defined as the proportion of expected HR response achieved, may be a clinical sign of sympathetic overactivity (10, 11), as with increased basal sympathetic outflow resting HR is elevated and HR reserve is narrowed. For adults with LLL who rely on HR response more than those with intact limbs, low chronotropic index may directly impact exercise capacity. While it is known that absolute HR response to exercise testing is higher among individuals with LLL when compared to controls without LLL (6) and that HR response to exercise testing is greater with higher LLL level (6), chronotropic index has not been evaluated in this clinical population.

While staged cardiopulmonary exercise testing (e.g., the Bruce treadmill protocol) is the preferred method for evaluating chronotropic index (11), such assessments are limited by clinical feasibility given equipment and staff training needs. Additionally, the proposed two-stage treadmill protocol (stage 1 = 0.58 m/s, 0.5% grade; stage 2 = 1.3 m/s, 1.5% grade) (11) implemented in chronotropic incompetence research requires achieving a fast gait speed consistent with the highest level of functional mobility after LLL (13), and therefore is not feasible for many adults with LLL. The 6-Minute Walk Test (6MWT) is a clinically-feasible alternative commonly used in rehabilitative settings that is valid and reliable for assessment of exercise capacity among adults with LLL (3, 14, 15). And, chronotropic response to the 6MWT is predictive of mortality in other clinical populations (16, 17). While preliminary evidence suggests individuals with below-knee LLL have a moderate HR response to the 6MWT, e.g., 72%–78% of age-predicted HR maximum (18), past research has not adjusted for resting HR, a key component of chronotropic index. Furthermore, factors associated with chronotropic index following LLL remain unknown.

To inform future clinical treatments seeking to improve exercise capacity after LLL, the two aims of this study were to evaluate predictors of chronotropic response to the 6MWT among adults with unilateral LLL and to determine if chronotropic index mediates the relationship between population-specific variables and 6MWT performance. We hypothesized that population-specific variables, such as higher LLL level and shorter time since LLL, would be associated with higher chronotropic index (i.e., greater HR response to exercise testing). Our secondary hypothesis was that chronotropic index would significantly mediate the relationship between LLL level and 6MWT distance.

## 2 Methods

Data were combined from two cross-sectional, observational research studies completed by the Delaware Limb Loss Studies laboratory between September 2013 and January 2024. Participants were recruited via the University of Delaware Interdisciplinary Limb Loss Clinic and local prosthetic clinics. Studies were considered for inclusion if protocols involved recording medication use and completion of the 6MWT. All participants signed written informed consent, and projects were approved by the University of Delaware Institutional Review Board for Human Subjects Research (project numbers: 531197 and 780745).

Participants were included in this study if they were ≥18 years of age, able to read and speak English, had undergone LLL at or above the ankle at least six-months prior, and had data of interest (i.e., self-reported medications, resting vital signs, and 6MWT). Participants were excluded if they did not use a prosthesis, had contralateral LLL at or above the ankle, were missing post-test vital signs, or had recorded vital signs outside of physiological limits. Duplicate participants across projects were eliminated; the most recent complete dataset was retained in the event of multiple evaluations.

### 2.1 Self-Report information

Demographics, medical history, and LLL-specific information, i.e., level, etiology, and time since initial LLL, were captured using standardized questionnaires and confirmed via interviews by trained examiners. Prosthesis use was characterized using the Houghton Scale of Prosthesis Use, a reliable (Intraclass Correlation Coefficient [ICC]_2,1_ = 0.96; 95% Confidence Interval [CI]: 0.92–0.97), valid 4-item measure where higher scores indicate greater prosthesis use and stability (19). Medication data was collected using a standardized medication sheet provided in advance of the evaluation and verified during the clinical examination for accuracy and completeness. In cases where no medications were listed and participants had signed a Health Insurance Portability and Accountability Act (HIPPA) consent form, data were extracted from the participant's prosthetic medical records if the data were available within 1 month of the onsite evaluation.

#### 2.1.1 Physical evaluation

Height and weight with the prosthetic device donned were collected during research evaluations. Individuals were seated for at least five minutes prior to the evaluation of resting vital signs. Resting HR was taken using a medical-grade pulse oximeter by a member of the research team or manually at the radial artery by a health professional (i.e., physician or physical therapist). Resting blood pressure was assessed with the participant seated and arm supported at heart level via an automated cuff (Connex ProBP 3400, Welch Allyn, Skaneateles Falls, New York) by a member of the research team, or manually using a sphyhmomanometer and stethoscope by a health professional.

Participants then completed the 10-Meter Walk Test (10MWT), a reliable (ICC_2,1_ = 0.96; 95% CI: 0.93–0.98) and valid assessment of gait speed among adults with LLL (20). The test was performed at self-selected, i.e., “normal, comfortable pace,” and fast, i.e., “as quickly but as safely as possible,” speeds over a 10-meter course with the central six meters timed. The first and final two meters were not timed to allow for gait speed acceleration and deceleration; assistive device use was permitted (21). Average gait speed across three trials per each condition was recorded. Faster gait speeds are indicative of better functional mobility (13).

#### 2.1.2 6-Minute Walk Test

Participants completed the 6MWT, a reliable (ICC_2,1_ = 0.97; 95% CI: 0.95–0.99) and valid measure of aerobic capacity following LLL (3). Participants were instructed to cover as much ground as possible in 6 min. While rest breaks were permitted, participants were advised the clock would not stop, and they should begin walking again as soon as they were able. Examiners refrained from conversation to avoid limiting performance (21) and walked slightly behind the participant to avoid pacing the individual. Upon completion of the 6MWT, participants returned to a seated position for vital sign measurement within one minute. To estimate effort, percent of age-predicted HR maximum achieved was calculated using the equation {%HR_max_ = post-test HR/[208 – (0.7*age)]} (22). Chronotropic index, which quantifies the proportion of expected HR increase and may indirectly indicate autonomic function, was calculated using the equation [Chronotropic Index = (posttest HR – resting HR)/(208 – (0.7*age) – resting HR)] (11, 23).

#### 2.1.3 Statistical analysis

Prior to statistical analysis, data was verified against deidentified evaluation forms, when available, and with the participant's prosthetic medical records when a HIPAA records release permitted use. As this was a secondary analysis of a cross-sectional dataset, an *a priori* power analysis was not completed. Statistical analyses were performed using SPSS Statistics version 29 (IBM, Armonk, NY). Descriptive statistics were calculated for demographics, LLL-related information, health history, and cardiovascular data. The Shapiro–Wilk test was used to determine normality of continuous data. Independent *t*-tests (parametric data) and Mann Whitney *U*-tests (nonparametric data) were used to evaluate between-group differences, i.e., below-knee vs. above-knee level, as appropriate. Unilateral below-knee LLL was inclusive of transtibial LLL and ankle disarticulation; unilateral above-knee LLL was inclusive of transfemoral LLL and knee disarticulations. Independent sample proportion and Chi-square tests were used for nominal data, as appropriate.

**Figure 1:**
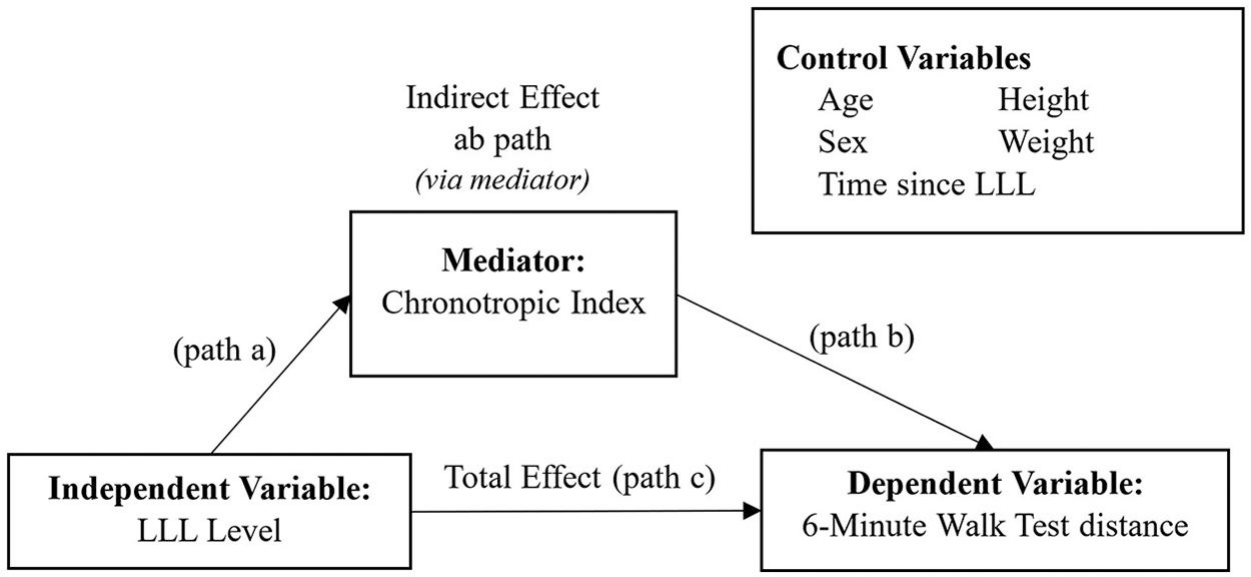
Explanatory diagram. A single mediator model showing an indirect effect of lower-limb loss (LLL) level on 6-Minute Walk Test performance (i.e., distance walked) through the mediator of chronotropic index, while controlling for suspected covariates.

Linear regression was used to identify predictors of chronotropic index. Covariates of age, sex, and diabetes were entered into Block 1; while LLL-specific variables (i.e., LLL level, time since LLL) were entered into Block 2. Etiology of LLL was not entered as a covariate due to significant overlap between self-reported diabetes and dysvascular LLL etiology. Beta-blocker use was entered into Block 3 to examine its unique influence, due to known suppression of HR (11). Model residuals were evaluated for normality using the Kolmogorov–Smirnov test. Outliers were critically evaluated and removed if data were suspect; assumptions for linear regression modeling were met. A mediation model (Figure 1) was then run using SPSS PROCESS Macro v4.2 to observe if chronotropic index mediated the relationship between LLL level and 6MWT performance (i.e., distance). Factors known to be associated with 6MWT distance, i.e., age (24), sex (24), height (24), weight (24), and time since LLL (25), were included as covariates. Significance was set at *α* = 0.050.

## 3 Results

**Figure 2:**
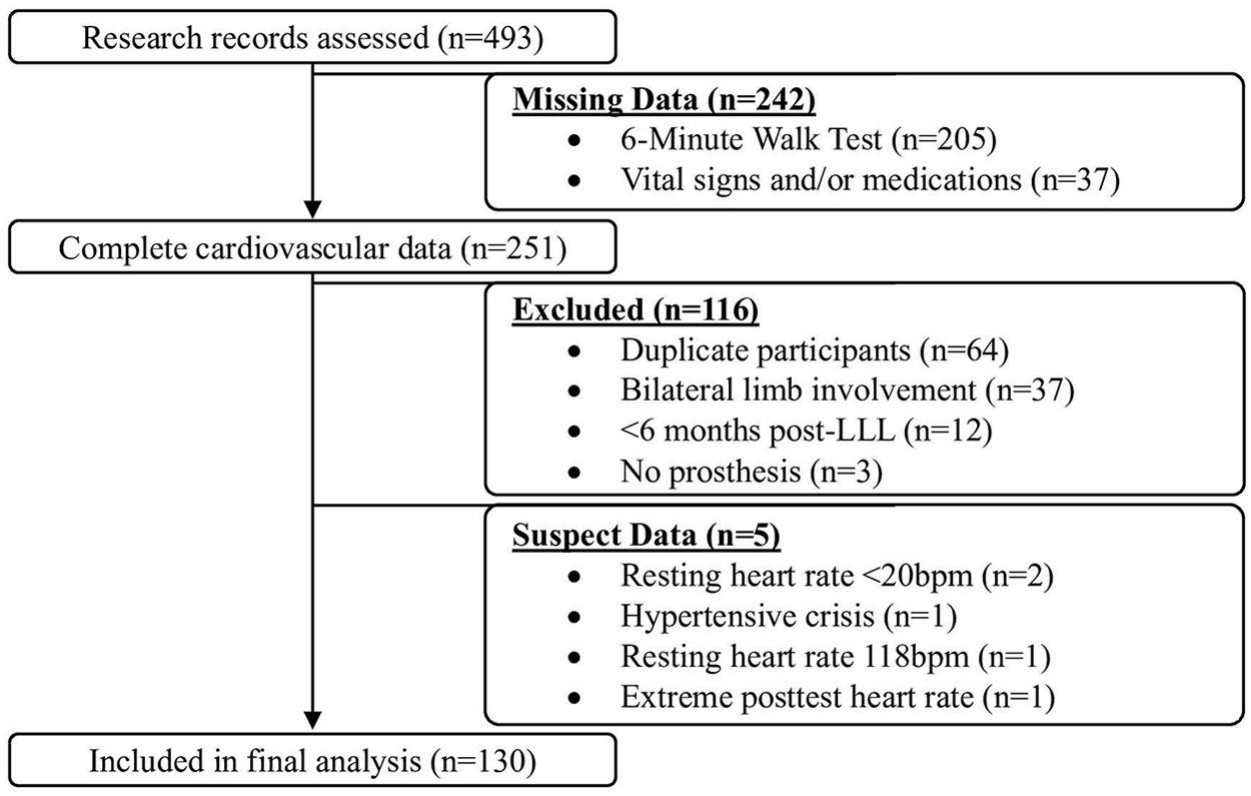
Participant inclusion flow diagram. *LLL*, *lower-limb loss; bpm*, *beats per minute.*

In total, 493 research records were considered for inclusion in the analysis. Of these, 205 individuals did not complete a 6MWT and 37 were missing post-test vital signs and/or medication information, resulting in 251 records with complete cardiovascular data. Of these, 64 were duplicate participants, 37 had bilateral limb involvement, 12 were less than six months post-LLL, and three lacked a prosthesis. Of the remaining 135 participants, five had suspect resting and/or post-exercise vital signs: two participants had resting HRs <20 bpm, one presented in hypertensive crisis with a resting blood pressure of 198/100 mmHg, one presented with an abnormally high resting HR at 118bpm, and one had a post-exercise HR >250% of their resting HR despite beta-blocker use. These cases were removed due to concerns about data validity. The final sample was comprised of 130 participants (Figure 2) of whom *n* = 95 (73.1%) had below-knee LLL and *n* = 35 (26.9%) had above-knee LLL.

Table 1 displays characteristics for the 130 participants. The average age of the sample was 57.3 ± 14.7 years. Participants with above-knee LLL weighed significantly less than peers with below-knee LLL (*p* = .033); etiology of LLL was also significantly different between groups (*p* = .021), with participants with above-knee LLL reporting a higher incidence of traumatic LLL and a lower incidence of dysvascular LLL. Overall, 49 participants (37.7%) reported diabetes, and 59 participants (45.4%) reported a history of smoking. Participants with above-knee LLL reported better health status, with fewer medications (*p* = .006), less frequent use of beta-blockers (*p* = .016), and lower diabetes prevalence (*p* = .034). Participants with below-knee LLL, however, were less likely to use an assistive device (*p* = .046) and had faster “fast” gait speeds (*p* = .003), indicating better mobility capacity.

**Table 1:** Between-group differences in sample characteristics based on lower-limb loss level.

Characteristic	Total Sample (*n* = 130)	Below-knee (*n* = 95)	Above-knee (*n* = 35)	*p*
**Demographics & Amputation Information**				
Age, *years*	57.3 ± 14.7	58.5 ± 14.9	54.0 ± 13.7	.123
Sex, *male*	95 (73.1%)	71 (74.7%)	24 (68.6%)	.482
Race, *White*	101/128 (78.9%)	73/94 (77.7%)	28/34 (82.4%)	.565
Height, centimeters**	175.3 (169, 180)	175.3 (169, 182)	172.7 (170, 178)	.144
Weight, kilograms**	86.4 (72.4, 109.1)	90.9 (75.7, 110.9)	75.0 (69.5, 97.7)	.033*
LLL Etiology				
*Dysvascular*	40 (30.8%)	34 (35.8%)	6 (17.1%)	.021*
*Trauma*	52 (40.0%)	34 (35.8%)	18 (51.4%)	
*Infection/Sepsis*	14 (10.8%)	13 (13.7%)	1 (2.9%)	
*Cancer*	12 (9.2%)	7 (7.4%)	5 (14.3%)	
*Congenital*	8 (6.2%)	6 (6.3%)	2 (5.7%)	
*Other*	4 (3.1%)	1 (1.1%)	3 (8.6%)	
Time since LLL, years**	7.2 (2.0, 18.7)	7.2 (2.7, 23.8)	7.2 (1.7, 18.4)	.612
Houghton Scale, *0–12*	10 (9, 12)	10 (9, 12)	10 (8, 12)	.150
**Health Status**				
History of Shortness of Breath, *yes*	18 (13.8%)	11 (11.6%)	7 (20.0%)	.218
Smoking History, *nonsmoker*	71 (54.6%)	53 (55.8%)	18 (51.4%)	.658
Diabetes, *yes*	49 (37.7%)	41 (43.2%)	8 (22.9%)	.034*
Cardiac Comorbidity, *yes*				
*Heart Disease*	18 (13.8%)	13 (13.7%)	5 (14.3%)	.930
*Hypertension*	63 (48.5%)	48 (50.5%)	15 (42.9%)	.438
*Cardiac Surgery*	20 (15.4%)	16 (16.8%)	4 (11.4%)	.448
Number of Medications, *n*	4 (1, 7)	5 (2, 8)	3 (1, 5)	.006*
*Beta-Blocker*	35 (26.9%)	31 (32.6%)	4 (11.4%)	.016*
Assistive Device Use, *none*	101 (77%)	78 (82.1%)	23 (65.7%)	.046*
Self-selected gait speed, m/s**	1.02 (.79, 1.21)	1.07 (.84, 1.22)	.93 (.73, 1.14)	.087
Fast gait speed, m/s**	1.40 (1.08, 1.67)	1.44 (1.19, 1.73)	1.28 (.85, 1.48)	.003*
**6MWT Data**				
Resting Vitals				
Heart rate, *beats/minute*	76.4 ± 13.4	76.0 ± 19.6	77.5 ± 11.8	.561
Systolic BP, *mmHg*	131.2 ± 18.9	130.6 ± 19.6	132.7 ± 16.9	.585
Diastolic BP, *mmHg*	77.9 ± 12.1	77.1 ± 12.5	79.9 ± 11.0	.246
Post-Test Vitals				
Heart rate, *beats/minute*	100.3 ± 22.4	97.3 ± 21.2	108.3 ± 23.9	.013*
%HR_max_	59.4 ± 12.7	58.9 ± 12.0	63.3 ± 13.6	.033*
Systolic BP, *mmHg*	154.9 ± 25.5	156.5 ± 26.7	149.4 ± 20.8	.289
Diastolic BP, *mmHg*	80.3 ± 14.0	80.5 ± 14.4	79.7 ± 12.8	.829
*Δ* Vital Signs				
Heart rate, beats/minute**	20 (8, 34)	18 (7, 29)	33 (13, 45)	.013*
Systolic BP, mmHg**	15 (6, 34)	18.5 (8, 37.3)	12 (0, 22)	.057
Diastolic BP, mmHg**	2 (−8, 8)	2 (−5.4, 9.5)	−2 (−10, 8)	.245
6MWT Distance, *meters*	381.2 ± 126.5	400.5 ± 126.4	328.9 ± 112.6	.004*
Estimated Max Distance, %	77.1 ± 15.7	76.8 ± 15.1	77.9 ± 17.3	.733
Chronotropic Index, %**	22.5 (10.0, 28.0)	21.1 (8.3, 32.3)	31.9 (14.2, 54.4)	.021*

* *p* < .050.

** Continuous data presented as median (25th, 75th percentiles) rather than mean ± standard deviation.

LLL, lower-limb loss; m/s, meters per second; BP, blood pressure; mmHg, millimeters of mercury; HR_max_, age-predicted heart rate maximum; 6MWT, 6-Minute Walk Test.

6MWT results are shown in Table 1. There were no significant differences in resting vital signs or blood pressure response (*p* > 0.05), but adults with above-knee LLL exhibited greater post-test HR (*p* = .013) and change in HR (*p* = .013), despite walking significantly shorter distances than peers with below-knee LLL, i.e., 328.9 ± 112.6 meters vs. 400.5 ± 126.4 meters, respectively (*p* = .004). Percent of age-predicted HR_max_ achieved was lower for adults with below-knee LLL when compared to peers with above-knee LLL, i.e., 58.9 ± 12.0% vs. 63.3 ± 13.6% (*p* = .033). Also, adults with below-knee LLL exhibited lower chronotropic indices than peers with above-knee LLL [21.1% [8.3%, 32.3%] vs. 31.9% [14.2%, 54.4%]; *p* = .021].

**Figure 3:**
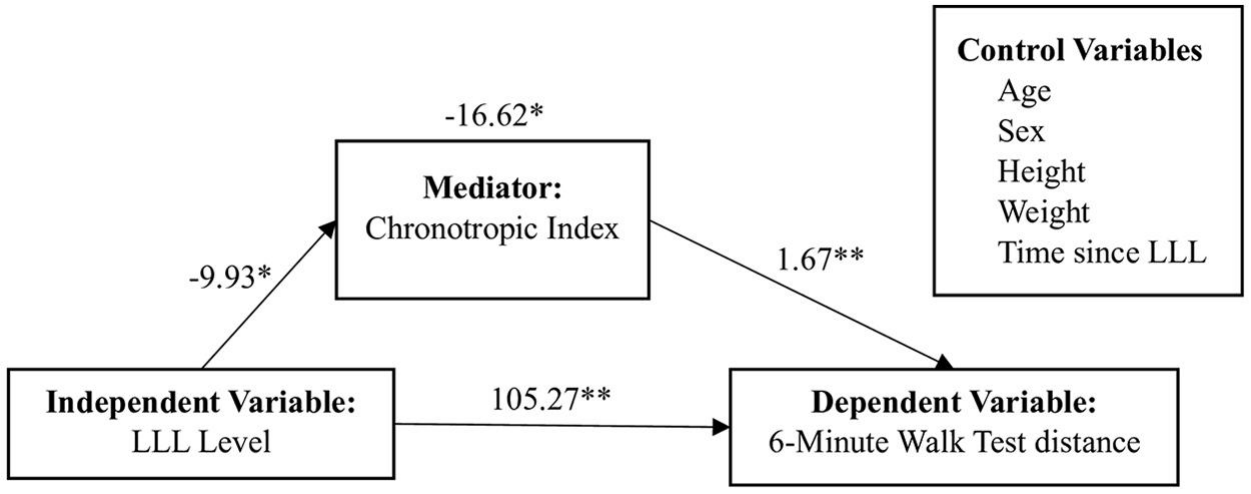
Mediation path model with parameter estimates (unstandardized beta coefficients). *p < .050; *p < 0.001.

Table 2 shows factors predictive of chronotropic index. LLL level and beta-blocker use significantly contributed to the model; both below-knee level LLL and beta-blocker use were independently associated with an 8% reduction in chronotropic index in Block 3. The model explained 6.3% of the variance in chronotropic index. Table 3 and Figure 3 show the result of the single mediation model. The total effect of LLL level on 6MWT distance was significant (*b* = 105.273, *p* < .001). LLL level was also significantly associated with chronotropic index (*b* = −9.929, *p* = .011), and chronotropic index was positively associated with 6MWT distance (*b* = 1.674, *p* < .001). The indirect effect through chronotropic index was statistically significant (indirect effect = −16.616, 95% CI: −35.057, −2.326); 15.78% of the total effect was explained by the indirect effect through the mediator. In addition to LLL level and chronotropic index, age (*p* < .001), height (*p* = .047), weight (*p* = .020), and time since LLL (*p* < .001) significantly contributed to the final model, which explained 43.5% of the variance in 6MWT distance.

**Table 2:** Linear regression model for predictors of chronotropic index (*n* = 130).

Block	Variable	B	SE	*β*	*P*	95% CI
1	Age, *years*	−.156	.120	−.119	.197	−.393, .082
	Sex, *male*	−.821	3.820	−.019	.830	−8.381, 6.739
	Diabetes, *yes*	1.178	3.615	.030	.745	−5.975, 8.332
	Adjusted r^2^ = −.010; *p* = .626					
2	Age, *years*	−.119	.118	−.091	.313	−.353, .114
	Sex, *male*	−.187	3.737	−.004	.960	−7.583, 7.210
	Diabetes, *yes*	1.401	3.712	.035	.707	−5.947, 8.748
	LLL level, *below*−*knee*	−9.411	3.830	−.218	.015*	−16.992, −1.830
	Time since LLL, *years*	−.133	.108	−.111	.220	−.346, .081
	Adjusted r^2^ = .037; *p* = .083					
3	Age, *years*	−.072	.119	−.055	.546	−.306, .163
	Sex, *male*	−.868	3.701	−.020	.815	−8.194, 6.458
	Diabetes, *yes*	2.226	3.683	.056	.547	−5.065, 9.518
	LLL level, *below-knee*	−8.046	3.835	−.187	.038*	−15.873, −1.453
	Time since LLL, *years*	−.116	.107	−.097	.278	−.327, .095
	Beta-blocker use, *yes*	−8.163	3.895	−.189	.038*	−15.873, −1.453
	Adjusted r^2^ = .063; *p* = .029*					

B, unstandardized beta coefficient; *β*, standardized beta coefficient; SE, standard error; CI, confidence interval; LLL, lower-limb loss.

* *p* < .050 .

**Table 3:** Mediation analysis of lower-limb loss level on 6-Minute Walk Test distance through chronotropic index (*n* = 130).

Pathway	B	SE	*p*-value	95% CI		BC 95% CI	
				Lower	Upper	Lower	Upper
LLL level, *below-knee* (IV)							
IV to mediator (a path)	−9.929	3.856	.011*	−17.563	−2.295		
Total effect (c path)	105.273	20.572	<.001**	64.548	145.998		
Chronotropic Index (M)							
Direct effect (b path)	1.674	.469	<.001**	.746	3.328		
Indirect effect (ab path)	−16.616	8.395				−35.057	−2.326
Partial effect of covariates							
Age, *years*	−3.254	.610	<.001**	−4.461	−2.406		
Sex, *male*	24.710	25.183	.328	−25.123	74.541		
Height, *cm*	2.530	1.256	.047*	.038	5.021		
Weight, *kg*	−.995	.424	.020*	−1.834	−.157		
Time since LLL, *years*	1.896	.559	<.001**	.791	3.002		
Model Summary (R^2^) = 0.435; *p* < 0.001**							

Dependent variable in the model: 6MWT Distance.

B, unstandardized beta coefficient; SE, standard error; CI, Confidence Interval; BC, bias corrected; LLL, lower-limb loss; IV, independent variable; M, mediator.

* *p* < .050;.

** *p* < .001.

## 4 Discussion

To our knowledge, this study is the first among adults with LLL to (i) identify predictors of chronotropic index and (ii) evaluate the role of chronotropic index as a mediator between LLL level and exercise capacity. Both LLL level and beta-blocker medication use predict chronotropic index, and findings suggest that chronotropic index partially mediates the relationship between LLL level and exercise capacity, as estimated with the 6MWT. Specifically, participants with below-knee LLL demonstrated reduced chronotropic response despite better 6MWT performance than peers with above-knee LLL, potentially due to level-specific differences in energetic cost of ambulation (26). Chronotropic index was independently associated with 6MWT distance, after controlling for known covariates. This suggests cardiovascular health, not balance and strength alone, influences mobility after LLL (27, 28). While cardiorespiratory fitness is mentioned in post-LLL rehabilitation protocols (29), in practice, current rehabilitation practices may be insufficient to improve cardiorespiratory fitness. Only half of individuals in inpatient prosthetic rehabilitation are exposed to sufficient physical strain to maintain pre-admission level of fitness, i.e., 40% of HR reserve for 30 min per day (30). Importantly, no post-acute amputation guidelines for cardiovascular fitness training exist, though resistance training has recently gained attention (31). Interventions to improve cardiorespiratory fitness via targeting of chronotropic index may be indicated to improve mobility and exercise capacity after LLL.

The average percentage of age-predicted HR maximum attained during the 6MWT reinforces that the 6MWT is a moderate-intensity submaximal test among adults with LLL, as our sample reached, on average, 59.4% (±12.7%) of their age-predicted HR maximum at 6MWT cessation. This is notably lower than the 70.2% (±12.3%) reported by Younesian et al. (32), despite longer distances walked in our clinical care-seeking sample, i.e., 381.2 ± 126.5 meters vs. 321.9 ± 128.0 meters, respectively. The lower HR response observed in our sample was potentially due to differences in sample characteristics, as Younesian and colleagues evaluated individuals being discharged from a six-week inpatient rehabilitation (acute LLL) with predominantly dysvascular LLL (32), while our sample consisted of community-dwelling adults at least six-months post-LLL (post-acute) with diverse LLL etiologies. Longer time since LLL is associated with greater self-reported mobility and longer 6MWT distance (25), which may reflect enhanced confidence or competence with prosthesis use relative to the acute training period; however, suppression of HR response during maximal testing suggests LLL-related reduction in chronotropic index could result from gradual adaptation to amputation, and may not be present in the acute rehabilitative phase. Alternatively, the inclusion of individuals on beta-blockers in the present study may influence differences in results, as Younesian et al. excluded individuals with cardiorespiratory problems, which may include use of cardioprotective medications, but medication use was not reported (32). Methodological differences may also contribute to the lower HR response seen in our sample, as our post-test HR was taken seated immediately after testing, while Younesian and colleagues utilized a HR monitor to capture instantaneous peak HR during testing (32).

Median chronotropic index was 22.5%, which is comparable to results observed among adults with chronic obstructive pulmonary disorder who were tested in the same manner (23). LLL level significantly predicted chronotropic index; below-knee LLL was associated with an 8% reduction in chronotropic index compared to above-knee LLL, suggesting reduced ability to access HR reserve (11). Prior research indicates adults with above-knee LLL exhibit an exacerbated HR response to graded exercise testing when compared to peers with below-knee LLL (6), likely due to increased energetic demands of prosthetic ambulation with higher LLL level (26). The increased cardiac needs for daily ambulation of individuals with more proximal LLL may be protective against chronotropic incompetence due to cardiac conditioning at lower absolute workloads. Alternatively, higher prevalence of diabetes and/or elevated body weight noted among adults with below-knee LLL as compared to above-knee LLL may contribute to lower chronotropic index, as body mass is known to be associated with reduced heart rate response to exercise among adults with diabetes (33), and chronotropic impairment is a known complication of diabetes (33). Further research is needed to elucidate if reduced chronotropic response to submaximal exercise testing among adults with below-knee LLL relative to higher amputation levels is related to hemodynamic disruption specific to LLL level, or results from the higher prevalence of comorbidity seen in this sample.

Beta-blocker use was also associated with an 8% reduction in chronotropic index, after considering covariates. Beta-blockers are prescribed for cardiovascular conditions, such as hypertension and angina, and attenuate HR at rest and during exercise via inhibition of beta-receptors, which raises clinical concerns about the potential adverse effects of beta-blocker use on exercise capacity (34). However, a recent study of 42,771 adults referred for cardiopulmonary exercise testing shows that while beta-blocker use reduces HR response, exercise capacity is not impaired, as the body adapts by increasing stroke volume (35). As adults with LLL exhibit suppressed stroke volume during exercise secondary to LLL-induced reduction in venous return due to the loss of the lower-limb and thus impaired skeletal muscle pump (6), findings may raise clinical concerns regarding the use of rate-limiting chronotropic medications, such as beta-blockers, following LLL.

Chronotropic index partially mediated the relationship between LLL level and 6MWT distance. There is a significant direct effect of LLL level and 6MWT distance, meaning below-knee LLL was associated with a significantly further 6MWT distance compared to above-knee LLL, which is consistent with prior literature (36). The direct associations between chronotropic index and exercise capacity may reflect inability to sufficiently increase cardiac output in participants with low chronotropic index, which has previously been noted during graded exercise testing among adults with LLL (6). There is a significant negative indirect effect of LLL level on 6MWT distance through chronotropic index, which suggests that chronotropic index may suppress the direct effect of LLL level on 6MWT distance. Thus, interventions to improve chronotropic index, e.g., exercise training (11), may be indicated for adults with below-knee LLL who present with low chronotropic response to the 6MWT to improve performance.

Beyond associations with cardiovascular disease mortality, attenuated chronotropic response to exercise may be a marker of autonomic imbalance. Findings among 172 adults without structural cardiovascular disease show low chronotropic response to bicycle ergometry is associated with abnormal HR variability metrics during exercise (37); heart rate did not increase despite sympathetic nervous system activation in that sample, suggesting autonomic imbalance. Similar findings have been noted among individuals with Parkinson's disease (38). As preliminary evidence suggests below-knee LLL is associated with impaired autonomic function (39), further research is needed to confirm the presence and impact of autonomic imbalance on exercise capacity after LLL. Studies evaluating the impact of coordinative (e.g., dance, Tai Chi) and endurance training on chronotropic index following LLL may be warranted, as these modalities have a greater effect on cardiac autonomic control than other forms of exercise (e.g., resistance training) (40).

### 4.1 Study limitations

Our study was limited by use of immediate post-test HR and lack of standardization of equipment used to assess heart rate (e.g., radial pulse, pulse oximeter) over the 10 + year duration of the study, which may introduce unnecessary variability in measured HR. Future use of continuous HR monitors or electrocardiography (ECG) to capture resting and peak HR, as well as to track HR recovery during and after exercise testing may enable more thorough assessment of chronotropic function (41). And, as ECG was not used, the influence of arrythmia on chronotropic index cannot be determined. Additionally, chronotropic incompetence was unable to be assessed, as incompetence is diagnosed using maximal exercise testing. Maximal exercise testing is also recommended for future research to evaluate if individuals with below-knee limb loss do not need to access as much of their cardiac reserve as those with above-knee limb loss to complete the 6MWT, which would result in lower chronotropic indices despite better cardiovascular health. Further, results should only be generalized to community-dwelling adults with unilateral below-knee or above-knee LLL who are at least six months post-LLL. Results may not apply to pediatric populations, those with bilateral LLL, and/or individuals in acute rehabilitation. Lastly, while findings may inform future prospective studies, the cross-sectional design precludes causative analysis. For example, while increased weight was identified as a predictor of poorer 6MWT performance, it is unclear whether increased weight decreases exercise capacity, or if increased weight results from lower physical activity and cardiovascular deconditioning.

## 5 Conclusion

Chronotropic response to the 6MWT may represent a clinically meaningful marker of exercise capacity among adults with LLL. Findings suggest beta-blocker use may be linked to poor chronotropic response after LLL. As chronotropic index mediates the relationship between LLL level and exercise capacity per 6MWT performance, prioritization of interventions targeting cardiac autonomic control may be indicated to improve exercise capacity following LLL.

## Data Availability

The raw data supporting the conclusions of this article will be made available by the authors, without undue reservation.
